# Generation and characterization of infectious molecular clones of transmitted/founder HIV-1 subtype C viruses

**DOI:** 10.1016/j.virol.2023.04.001

**Published:** 2023-06

**Authors:** Bonisile Luthuli, Kamini Gounder, Martin J. Deymier, Krista L. Dong, Alejandro B. Balazs, Jaclyn K. Mann, Thumbi Ndung'u

**Affiliations:** a Africa Health Research Institute, Durban, South Africa; b HIV Pathogenesis Programme, The Doris Duke Medical Research Institute, University of KwaZulu-Natal, Durban, South Africa; c The Ragon Institute of MGH, MIT and Harvard University, Cambridge, MA, USA; d Division of Infection and Immunity, University College London, London, UK

**Keywords:** HIV-1, Subtype C, Transmitted/founder, Infectious molecular clones

## Abstract

The genetic diversity of HIV impedes vaccine development. Identifying the viral properties of transmitted/founder (T/F) variants may provide a common vaccine target. To study the biological nature of T/F viruses, we constructed full-length clones from women detected during Fiebig stage I acute HIV-1 infection (AHI) from heterosexual male-to-female (MTF) transmission; and clones after one year of infection using In-Fusion-based cloning. Eighteen full-length T/F clones were generated from 9 women and six chronic infection clones were from 2 individuals. All clones but one were non-recombinant subtype C. Three of the 5 T/F clones and 3 chronic clones tested replicated efficiently in PBMCs and utilised CCR5 coreceptor for cell entry. Transmitted/founder and chronic infection clones displayed heterogenous in vitro replicative capacity and resistance to type I interferon. T/F viruses had shorter Env glycoproteins and fewer N-linked glycosylation sites in Env. Our findings suggest MTF transmission may select viruses with compact envelopes.

## Research highlights

• We generated infectious molecular clones of HIV-1 subtype C transmitted/founder (T/F) and chronic infection variants.
• The HIV-1 subtype C infectious clones display heterogeneity in in vitro replicative capacity and resistance to type I interferon.
• T/F clones predominantly use the CCR5 coreceptor; shorter Env variable regions and fewer glycans compared to chronic viruses.

## 1 Introduction

The human immunodeficiency virus type 1 (HIV-1) pandemic is characterised by enormous genetic diversity due to its high error prone viral reverse transcriptase (RT) (Roberts et al., 1988). The genetic diversity of HIV-1 stems from the accumulation of mutations in the viral genome and is manifested in chronically infected individuals as a complex quasispecies of many genetically distinct viral genomes (Maldarelli et al., 2013). This diversity is one of the major obstacles to the development of a vaccine (Gao et al., 2005; McBurney and Ross, 2008). However, during HIV transmission a genetic bottleneck exists such that only one or a few genetic variants from a chronically infected donor establish infection in the newly infected host (Miller et al., 2005; Wolinsky et al., 1992; Zhang et al., 1993). This creates a window of opportunity to disrupt HIV transmission. Understanding how the immune system responds to variants that are preferentially transmitted may be key to the development of effective immune-based or other prophylactic interventions against HIV-1.

The observation that ∼80% of heterosexual transmission events of HIV-1, are established by a single transmitted/founder (T/F) virus (Shaw and Hunter, 2012) led to the hypothesis that certain genetic and phenotypic traits are selected for transmission. Genetic comparisons of the *env* sequences of subtype A and C T/F viruses to those of transmitting partners or chronic infection viruses show that shorter variable loops and fewer potential N-linked glycosylation sites (PNGs) are selected for in T/F viruses, although this was not the case in subtype B viruses (Chohan et al., 2005; Derdeyn et al., 2004; Sagar et al., 2009). A comprehensive phenotypic comparison between the T/F and chronic viruses demonstrated that T/F viruses have enhanced infectivity, incorporate higher Env content per viral particle and are relatively more resistant to inhibition by type I interferon (IFN-α) than chronic viruses (Parrish et al., 2013). A recent study generated subtype C full-length clones near the time of transmission from heterosexual transmission pairs from plasma and reported that T/F viruses were more consensus-like but were not more resistant to IFN-α as compared to non-transmitted viruses (Deymier et al., 2015), contradicting earlier studies. In a mother-to-child (MTC) transmission setting of subtype C viruses, consensus-like viruses with low replicative capacity were preferentially transmitted (Naidoo et al., 2017).

The contradictory findings on T/F traits prompted us to generate full-length genome infectious molecular clones (IMCs) of HIV-1 viruses in order to investigate the biological characteristics of HIV transmission in the South African context. Moreover, we reasoned that there may be other biological characteristics of T/F viruses and replication-competent viral clones of these variants are needed to aid the identification and detailed characterization of such novel properties. Studies to enable their detailed biological characterisation of T/F viruses have been impeded by the limited availability of full-length IMCs of these variants. South Africa carries a significant share of the global HIV burden, with an HIV prevalence of 18.9%. In South Africa, young women are disproportionately infected with HIV compared to their male counterparts (Mabaso et al., 2019; Shisana et al., 2005, 2010). Evidence suggests that the selection bias is less stringent in male to female (MTF) than female to male (FTM) transmission, possibly due to differences in physical and immunological properties of the genital mucosa between men and women (Carlson et al., 2014). Little is known about how T/F virus phenotypic properties identified in previous studies compare to T/F virus phenotypic properties observed in women infected via MTF transmission. In addition, no study has reported generation of subtype C IMCs of T/F viruses derived from as early as Fiebig stage I of AHI.

In this study, we generated IMCs of subtype C T/F viruses derived near the time of transmission in young women in South Africa and characterised their genotypic and phenotypic properties to further elucidate the biology of HIV-1 transmission in this population. In this report, we describe the generation of 18 T/F and 6 subtype C chronic infection full-length genome clones from 9 to 2 individuals, respectively. We analysed their ability to infect immortalized cell lines and primary cells, coreceptor tropism, and sensitivity to type I interferon (IFN-α2a and IFN-β) inhibition. Our results demonstrated that T/F viruses display a wide range in particle infectivity. The clones were able to propagate in peripheral blood mononuclear cells (PBMCs) as well as U87.CD4.CCR5 cells and resisted IFN-α2a more than IFN-β. Comparisons of T/F-derived viruses with chronic circulating viruses revealed a preference for compact envelopes with short Env variable regions and fewer N-linked glycosylation sites at transmission.

## 2 Materials and methods

### 2.1 Clinical samples

The ten participants investigated in this study were selected from the FRESH cohort of acutely infected individuals (Dong et al., 2018). FRESH is an observational, prospective cohort study based in Umlazi township, Durban, South Africa. In this cohort, young women at high risk of acquiring HIV, between the ages of 18–23, were enrolled in a job-skills poverty-alleviation programme that was integrated with a clinical study that included monitoring twice a week by finger prick blood draw for HIV-1 RNA using a PCR assay (NucliSens EasyQ v2.0 assay; BioMérieux, Marcy l’Etoile, Switzerland). As a result of this frequent testing strategy, the majority of individuals with acute HIV-1 infection are identified within 4 days of their last negative HIV-RNA during Fiebig stage I AHI. Participants with a detectable HIV-1 RNA were called in for immediate sample collection, confirmatory HIV-RNA and serial AHI blood markers. All T/F viruses studied were collected from participants detected during Fiebig stage I AHI. The median log viral load at acute infection was 5.405 log10 copies/ml. The CD4^+^ T cell counts ranged from 204 to 1025 cells/mm^3^ (median 460 cells/mm^3^) (Table 1). Samples were collected 1–4 days after detection of AHI, with a median of 2 days.

**Table 1:** Clinical profiles of study participants at sampled time points.

PID	No. of days from detection	Year of sampling	Log viral load (copies/ml)	CD4^+^ T-cell count (cells/μl)	Subtype
**036**	3	2013	6.38	204	C
**036**	332	2014	5.11	305	C
**039**	1	2013	5.6	432	C
**079**	3	2013	5.38	637	C
**079**	329	2014	4.08	532	C
**079**	687	2015	4.67	405	C
**102**	3	2013	5.43	814	C
**102**	711	2015	5	589	C
**186**	4	2013	6.8	306	C
**186**	745	2015	5.04	262	C
**198**	1	2013	6.57	367	C
**479**	1	2015	4.15	1025	C
**498**	1	2015	4.73	631	C
**499**	1	2015	4.72	738	C
**527**	3	2015	4.58	685	AC

The study was approved by the Biomedical Research Ethics Committee of the University of KwaZulu-Natal (BREC REF: BE699/18) and all study participants provided written informed consent.

### 2.2 Amplification and cloning of the viral molecular clones

Participant plasma samples were thawed at room temperature. HIV RNA was extracted, using the QIAamp viral RNA extraction kit (Qiagen, Valencia, USA) from 140 μL of the participants’ plasma according to the kit instructions. Complementary DNA (cDNA) was synthesized from RNA using the Superscript IV reverse transcriptase enzyme (Invitrogen, Life Technologies, MA, USA).

The resultant cDNA product was used as the template for first round PCR amplification. Two first round PCR reactions were performed to amplify the HIV-1 genome into half genome amplicons. For amplification of the first half genome (5′ half genome); the PCR reaction mix were performed in 1X Q5 reaction buffer, 0.25 mM of deoxyribonucleotide triphosphate (dNTPs), 0.8 μM primers and 0.02 U/μl High Fidelity Q5 Hot Start DNA polymerase (New England Biolabs, Ipswich, USA) in a total reaction volume of 20 μL. For amplification of the first half of the genome, the first-round primers were R_For_1 and Int_Rev_1 and second round primers were R_For_2 and Int_Rev_2 **(**Table S1**)**. For the second half of the genome (3′ half), the first-round primers were Int_For_1 and R_Rev_1 and second half second round primers were Int_For_2 and R_Rev_2. Cycling conditions for both PCRs were 98 °C for 30s, followed by 30 cycles of 98 °C for 10s, 60 °C for 30s, 72 °C for 3 min and 30s, with final extension at 72 °C for 2 min. The PCR products were loaded onto a 1% agarose gel and separated by electrophoresis at 100 V for 1 h to determine the presence of a 4.5 kb band.

### 2.3 Amplification of pHAGE lentiviral linearised vector

The pHAGE plasmid, containing HIV-1 subtype B U5 region and CMV-driven LTR was supplied by Alejandro Balazs (The Ragone Institute of Massachusetts General Hospital, Massachusetts Institute of Technology and Harvard University). The vector (pHAGE-wtCMV-DsRED express-BrCr1 ZsGreen-W plasmid) was used as a template to amplify a 2.6 kb region containing the HIV U5 region from Subtype B, an ampicillin resistance gene, origin of replication and CMV promoter. To produce the vector fragment for cloning, 1 ng of DNA in 1 μL was used as a template. The PCR reaction mix included 1X Q5 reaction buffer, dNTPs (10 mM each), 20 μM of both the forward primer Vec_For and the reverse primer Vec_Rev, High Fidelity Q5 Hot Start polymerase and distilled water was added up to a final volume of 50 μL. The samples were placed in a thermocycler and the reaction was run under the following conditions: 98 °C for 30s, 30 cycles for 98 °C for 10s, 69 °C for 30s, 72 °C for 2 min and 30s, and 1 cycle of 72 °C for 2 min. The PCR product was loaded onto a 1% agarose gel and migrated at 140 V for 50 min.

### 2.4 Cloning of HIV-1 half genome amplicons into a lentiviral vector

**Fig. 1:**
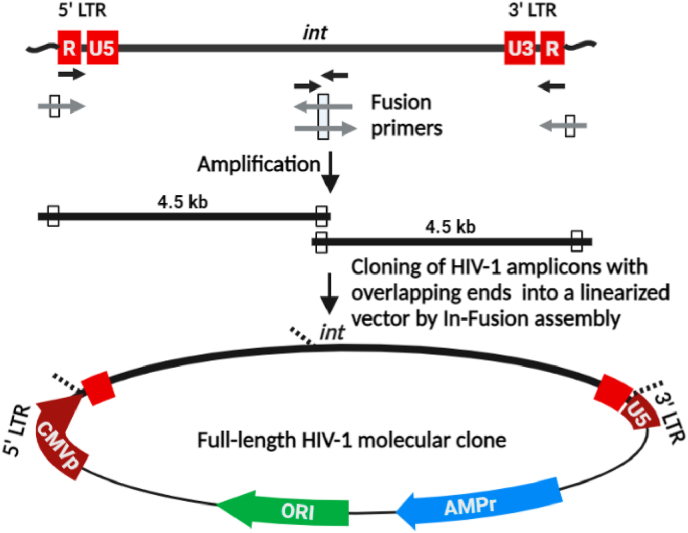
Diagram for the half-genome cloning strategy. Schematic representation of ligation independent fusion cloning for HIV-1. A viral RNA is depicted at the top of the cartoon. The figure is not drawn to the scale. The U3, R and U5 regions of the LTRs are shown in red. Arrows represent primer location and the orientation. The black arrows are used for the first-round and the grey arrows for the second-round amplifications. Note that only the second-round primers contain homologous bases for cloning (white boxes). The *IN* primers, in both orientations, are complementary to a highly conserved sequence within *IN*. The complete viral genome is amplified into two fragments and cloned into a vector in an In-Fusion reaction to generate full-length molecular clones. Broken lines indicate in-fusion sites.

In order to generate IMCs from T/F half genome amplicons, we utilised a ligation-independent cloning strategy using the Clontech In-Fusion HD system (Takara Bio, Kusatsu, Japan) to perform a three-piece DNA fusion reaction followed by transformation of high transformation efficiency chemically competent bacteria. The linearised vector contained matched 15 bp sequences at the ends while the half genome amplicons contained complementary 15 bp sequence at *integrase* (*IN*), Fig. 1. The two half genomes were purified and then cloned into the linearised lentiviral vector fragment produced in section 2.3 using an In-Fusion HD in a 15-min reaction to generate a 12 kb full-length molecular clone of the virus.

### 2.5 Generation of molecular clones from single genome amplified near full-length genome

Molecular clones from participants 036, 079 and 198 were generated based on previously described methods (Deymier et al., 2014). Briefly, viral RNA was extracted from plasma and converted to full-length cDNA. cDNA was serially diluted and subjected to nested PCR amplification with HIV-specific primers that yield 9-kb near full-length (NFL) amplicons. cDNA dilutions were tested to identify a dilution where ∼30% of wells were positive for amplification products. Patients LTR were amplified from genomic DNA and cloned into a pBluescript vector. In a 15-min Clontech In-fusion reaction patient-derived LTR and pBluescript and NFL DNA pieces were combined to form a full-length molecular clone.

### 2.6 Cells and cell lines

Human epithelial kidney 293 T (HEK293T) cells and TZM-bl cells were propagated in Dulbecco's modified eagle medium (DMEM) supplemented with 10% fetal bovine serum (FBS), and 50 IU/ml penicillin-streptomycin. U87 CD4 cells expressing chemokine receptors CCR5 or CXCR4 were maintained in DMEM supplemented with 10% FBS in medium containing 1 μg/ml of puromycin, 300 μg/ml G418, and 50 IU/ml penicillin-streptomycin. PBMCs from HIV-1 negative donors were cultured in RPMI 1640 supplemented with 10% FBS, 5 μg/ml phytohemagglutinin (PHA) and 20 IU/ml interleukin-2 (IL-2) for 48 h prior to infection.

### 2.7 Production of viral stocks from the molecular clones and viral infection assay

Viruses were generated by transfecting HEK293T cells using Mirus *Trans*-IT transfection reagent (Mirus Bio LLC, Wisconsin, USA). Cells seeded at 0.5 × 10^6^, in 6 well plates were transfected with a total of 2.5 μg of viral plasmid DNA. At 24-h post transfection, medium was removed and replaced with fresh DMEM. Cell culture supernatants were harvested at 48 h post transfection, clarified at 1800×*g* for 5 min and then frozen at −80 °C. The p24 concentration of viral stocks was evaluated using a commercial ELISA kit (Zeptometrix Corporation, NY). IMC-derived viruses with genetically intact genomes were tested further in a series of functional assays in which one representative T/F clone from each PID was chosen.

The median tissue culture infectious dose (TCID50) titers of viral stocks were determined by using TZM-bl cells. In a 96 well plate, 100 μL of DMEM growth media was added in each well, and viral sample (40 μL) was added to the wells of the first column in quadruplicates and serially diluted across the plate. Freshly trypsinized TZM-bl cells (10,000) were added to each well. To examine luciferase expression after 48 h, culture media (100 μL) was removed from each well and 100 μL of Steady-Glo substrate (Promega, Madison, USA) was added as per the manufacturer's recommendation. The plates were incubated for 2 min at room temperature to allow cell lysis. The cell lysate was transferred to a flat bottom black solid 96 well plates for measurement of luminescence, expressed as relative light units (RLU) using the GloMax Explorer plate reader (Promega, Madison, USA). Data was analysed in Microsoft Excel, where tissue infectious dose for each virus was determined by lowest dilution where 50% viral entry was achieved.

Viral infectivity was determined by infecting TZM-bl cells. In a 96-well plate, viral samples equivalent to 5 ng of p24 were added to 100 μL of DMEM growth medium per well in quadruplicate. Freshly trypsinized TZM-bl cells (10,000) were added to each well and incubated. After 48 h, culture media (100 μL) was removed from each well and 100 μL of Steady-Glo substrate was added as per the manufacturer's recommendation. The plates were incubated for 2 min at room temperature to allow cell lysis. The cell lysate was transferred to flat bottom black solid 96 well plates for measurement of luminescence. The threshold for viral infectivity was defined as 3 standard deviations above no virus control.

### 2.8 Determination of coreceptor usage

U87 CD4 cell lines with coreceptor CCR5 or CXCR4 were used. Viral stocks, 4000 TCID units each, were added to 0.5 × 10^6^ cells in a 6-well plate in DMEM medium supplemented with 10% FBS. After 16 h of incubation, residual virus was washed off, fresh media was added, and the cells were incubated. Culture supernatants were sampled periodically up to 14 days and viral infection of the target cells was monitored using p24 ELISA. On day 7, the cells were split, and the medium was removed from each well and washed with PBS. Trypsin (200 μl) was added to each well, cells were washed and resuspended in 5 ml in fresh DMEM growth media and plated in a T25 flask and incubated at 37 °C.

### 2.9 Determination of viral replication

Viral replication was assessed using methods based on those previously published (Kiguoya et al., 2017). Activated PBMCs (2 × 10^6^ cells) were infected with virus equivalent to 20 ng of p24 in a 6 well plate. After 16 h incubation, cells were washed 3 times and resuspended in fresh R10 with IL-2 and transferred to a new 6 well culture plate. Every 3 days, culture supernatants were collected for up to 13 days post infection. Virus released into the supernatant was quantified by HIV p24 antigen ELISA.

### 2.10 Determination of viral resistance to type I interferon

The sensitivity of the viruses to infectivity inhibition by type I IFN was assessed using methods adapted from those previously published (Fenton-May et al., 2013). In a 96-well plate, TZM-bl cells (1.5 × 10^4^ cells) were treated with either IFN-α2a (Sigma-Aldrich, St. Louis, USA) or IFN-β (Sigma-Aldrich, St. Louis, USA) at concentrations from 0 to 5000 IU/ml in DMEM growth medium. After 4 h of incubation, viral stocks of 1500 TCID units each were added to cells in 96-well plates with DMEM medium supplemented with 10% FBS. After 48 h the media (100 μl) was replaced with 100 μl of Steady-Glo substrate followed by 2 min of incubation. The cell lysate was transferred into a flat bottom black 96 well plates and read using GloMax Explorer plate reader (Promega). Data was analysed in Microsoft Excel and IFN sensitivity was determined by calculating half maximal inhibitory concentration (IC_50_) and viral infectivity at the highest IFN dose, expressed as residual infectivity (Vres) from a non-linear sigmoidal curve.

### 2.11 Sequencing and sequence analyses

We used more than 40 individual sequence primers spanning the entire length of the viral genome on both strands. Sequencing reactions were performed on the plasmid DNA. Overlapping contiguous sequences, for molecular clones, were obtained throughout the genome using the dye-terminator chemistry. Chromatograms from ABI Prism 377 DNA sequencer and Beckman sequencer were edited using Geneious v10.0.9 bioinformatics software (Biomatters, Auckland, NZ). Sequence contigs were assembled on Geneious to generate complete nucleotide sequences of the molecular clones.

Sequences of the newly derived viral clones were aligned with full-length reference sequences of several Group M viruses obtained from the Los Alamos sequence database (https://www.hiv.lanl.gov/cgi-bin/NEWALIGN/align.cgi, accessed in August 2021). HIV subtype confirmation was done using the recombinant identification program (RIP) (http://www.hiv.lanl.gov/content/sequence/RIP/RIP.html, accessed July 2021) and Rega HIV Subtyping tool (https://www.genomedetective.com/app/typingtool/hiv, accessed September 2022). Maximum-likelihood phylogenetic trees were generated using MEGA-X with 100 bootstrap replicates.

Coreceptor usage and third variable region sequence net charge were predicted on the basis of the V3 loop sequence using an online tool; webPSSM (https://indra.mullins.microbiol.washington.edu/webpssm/, accessed 27 June 2021) The number of potential N-linked glycosylation sites (PNGs) in envelope were determined using the N-Glycosite tool provided in the Los Alamos National Laboratory database (https://www.hiv.lanl.gov/content/sequence/GLYCOSITE/glycosite.html, accessed February 2023).

### 2.12 Nucleotide sequence accession numbers

The sequences have been submitted to GenBank with accession numbers ON862672-ON862692.

### 2.13 Statistical methods

All statistical analyses were performed in GraphPad Prism 9.1.2.

Comparative analyses were performed using the Mann-Whitney *U* test. The significance cut off for all statistical analyses was p < 0.05.

## 3 Results

### 3.1 Generation of molecular clones of T/F viruses from South Africa

With an objective to generate IMCs of T/F virus, we developed a cloning strategy that would favour amplification and cloning of full-length subtype C viruses. The strategy involved amplification of half genomes from viral RNA, obviating the need for full-length genome amplification, which is usually challenging because of the two identical long terminal repeats (LTRs) on either side of the genome. The cloning strategy depends on the use of primers with 15 bp overlaps in the LTRs and in the *IN* gene of HIV-1 (Fig. 1). The overlapping primers permit amplification of HIV genome into two fragments which, when assembled can generate a full-length viral clone. The region of *IN* selected for primer binding is highly conserved among all the major viral subtypes of HIV-1. Two external primers were designed to bind the 5′ and 3’ ends of the LTRs, respectively. Using the two primer pair combinations (a) R_For_1 and Int_Rev_1 and (b) Int_For_1 and R_Rev_1, the HIV-1 viral genome can be amplified in two fragments, N-terminal and C-terminal, of approximately 4.5 kb length each.

**Fig. 2:**
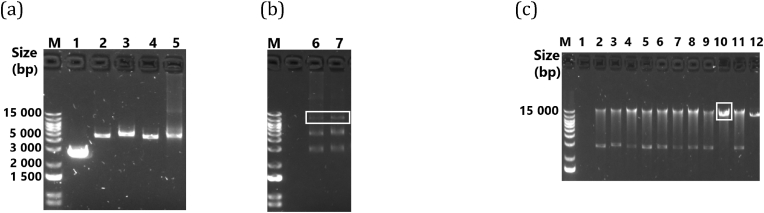
Construction of molecular clones. (a) Agarose gel electrophoresis images showing a gel purified linearised vector piece (lane 1), second PCR products of ∼4.5 kb from PID 527 (lanes 2–3) and PID 039 (lanes 4–5). The two 4.5 kb amplicons were cloned with the vector piece that compliments the missing LTR portions. (b) The resultant three-piece DNA infusion products for PID 527 (lane 6) and PID 039 (lane 7) are shown by a white rectangle which indicates the presence of a 12 kb target DNA. (c) Following transformation the 12 kb plasmid DNA was purified as shown by a white box (lane 10).

Following PCR amplification of the half genomes and the linearised vector, the PCR products were analysed for the correct size using agarose gel electrophoresis before cloning. Representative two half genome DNA amplicons and a 2.6 kb linearised vector piece are shown in Fig. 2a. Upon confirmation of size, the amplicons together with the linearised vector were fused in a 15-min in-fusion reaction to form a 12 kb molecular clone (Fig. 2b). A plasmid DNA of full-length molecular clone of a T/F virus was later purified following bacterial transformation Fig. 2c. Using this strategy, we generated full-length molecular clones from 7 acutely infected individuals.

Additional molecular clones were generated by single genome amplification of near full-length HIV-1 genome and cloned into a pBluescript vector as described previously (Deymier et al., 2014). We amplified near full-length (NFL) single genomes by limiting dilution PCR from patient plasma collected during acute infection and from plasma collected during chronic infection. With this method, we generated molecular clones from 3 individuals— 8 T/F clones from 2 individuals and 6 molecular clones at 1 year post infection from 2 individuals (Table 2).

**Table 2:** Number of full-length HIV-1 molecular clones generated.

Cloning method	Participant ID	Days post detection	Number T/F of clones	Number of chronic clones
**Bulk PCR method**	039	1	**1**	
	102	3	**1**	
	186	4	**1**	
	479	1	**1**	
	498	1	**1**	
	499	1	**1**	
	527	3	**3**	
**SGA method**	036	332		**4**
	079	3	**2**	
		329		**2**
	198	1	**6**	
**Total number**			**18 from 9 PIDs**	**6 from 2 PIDs**

### **3.2** Viral molecular clones from 8 individuals were infectious

**Fig. 3:**
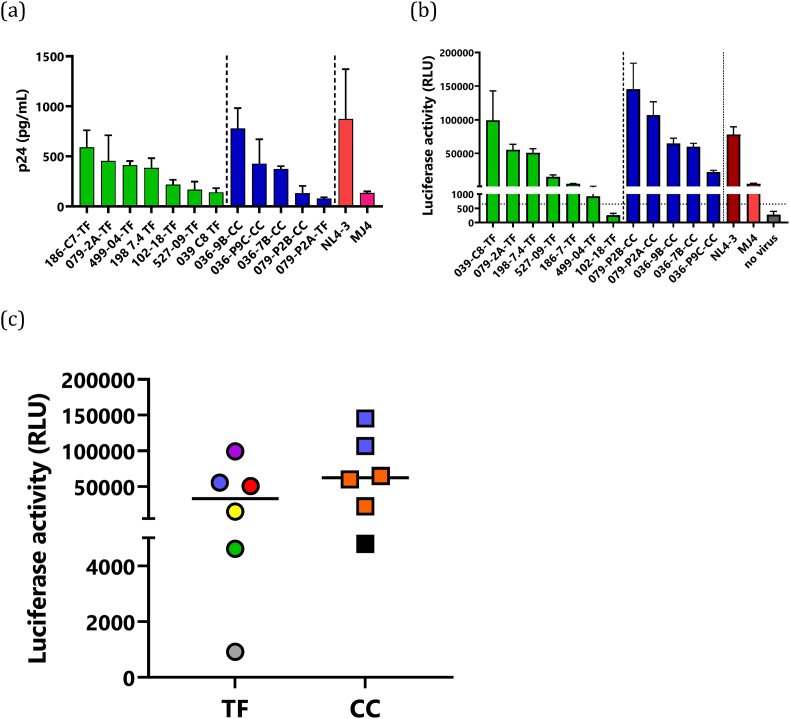
Analysis of viral production and infectivity of molecular clones. (a) 12 patient-derived molecular clones were individually transfected into HEK293 cells. Reference molecular clones, MJ4 and NL4-3, served as positive controls. Cell culture supernatant was collected 48 h following transfection and p24 production was quantified using commercial antigen ELISA. Viral production of each virus variant was measured for 2 separate virus stocks generated from 2 independent 293T transfections, where error bars represent the standard error of the mean. (b) The ability of virus produced by the molecular clones to infect TZM-bl cells was quantified. 1 × 10^4^ cells were infected with viral supernatant equivalent to 5 ng of p24. Infected cells were monitored for luciferase expression 48 h following infection. The black dotted lines represent the threshold line for infectivity defined as 3 standard deviations above no virus control relative light unit. Viral infectivity of each virus variant was measured in quadruplicates in 2 separate experiments, where error bars represent the standard error of the mean. (c) Comparison of luciferase activity of 6 T/F viruses (039-C8-TF, purple; 079-2A, blue; 186-C7-TF, green; 198 7.4-TF, red; 499-04-TF, grey; 527-09-TF, yellow) and 6 chronic infection viruses (036-7B-CC, 036-9B-CC and 036-P9C-CC, orange; 079-P2A-CC and 079-P2B, blue; MJ4, black). T/F viruses (circles) and chronic infection viruses (squares) showed heterogenous luciferase activity. Viruses from the same participant; PID 079 are shown in blue. Statistical comparisons are not shown because the clones are derived from a very limited number of participants.

Next, we tested for the expression of the Gag p24 protein following transfection of HEK293T cells with the purified plasmid DNA of each molecular clone separately. All molecular clones as well as the reference subtype C molecular clone MJ4 produced p24 suggesting virus production in 293T cells. For the T/F-derived clones, p24 production ranged from 140 ng/ml to 588 ng/ml. Among the chronic derived clones, p24 production ranged from 79 to 778 ng/ml with the molecular clones from PID 036 resulting in the highest p24 concentrations. We noticed that certain clones, 039-C8-TF, 079-P2A-CC, 079-P2B-CC, and 527-09-TF repeatedly showed low p24 production below 200 ng/ml. The p24 concentration of the virus stocks was comparable to that of a previously described subtype C IMC, MJ4, which had a mean p24 concentration of 133 ng/ml, and a subtype B IMC, NL4-3, which had a mean p24 concentration of 873 ng/ml (Fig. 3a).

To detect virus infectivity, we determined the expression of Tat induced luciferase in TZM-bl cells under the control of HIV-1 LTR. In cases where more than one T/F clone was generated per individual, we tested one representative clone for infectivity. Eight molecular clones expressed luciferase indicative of successful viral entry, reverse transcription and productive induction of Tat signalling (Fig. 3b**).** Both T/F and chronic infection derived viruses showed variable infectivity after 48 h. One clone 102-18-TF showed luciferase expression below the infectivity threshold after 48 h. We confirmed infectivity of virus stocks on TZM-bl cells where 7 T/F and 5 chronic-derived clones were tested. Six of the seven T/F from seven individuals and five chronic infection clones from two individuals were infectious. The median infectivity values were similar between both groups: 32857RLU for T/F, 62347 RLU for chronic infection viruses (Fig. 3c**)**, however statistical comparisons were not performed since the tested clones were not independent, particularly the chronic infection clones that were generated from only 2 participants. Overall, these results indicate that the infectivity of both T/F and chronic infection subtype C clones is heterogenous but generally comparable to that of the prototype subtype C infectious molecular clone, MJ4. However, to accurately determine whether T/F or chronic infection viruses have enhanced infectivity will require generation of more clones from diverse participants to enable independent statistical comparisons.

### 3.3 Sequence analysis of the full-length genomes

The reading frames of all the structural, regulatory and accessory proteins were evaluated. From the 21 molecular clones sequenced, 17 clones from 7 participants had preserved proteins and 4 clones from 4 participants had genetic defects. We identified premature stop codons in 479-11-TF, 498-03-TF, 079-8A-TF and 036-1B-CC (Table 3).

**Table 3:** Sequenced molecular clones with defective genomes.

PID	Clone name	Genome	Defect location	Infectivity
036	036-1B-CC	Defective	***Pol*** (1 bp insertion in integrase); Env (1 bp deletion)	**Not infectious**
	036-7B-CC	Intact		Infectious
	036-9B-CC	Intact		Infectious
	039-P9C-CC	Intact		Infectious
039	039C8-TF	Intact		Infectious
079	079-2A-TF	Intact		Infectious
	079-8A-TF	Defective	***Vif* (**7 pb deletion)	Infectious
	079-P2A-CC	Intact		Infectious
	079-P2B-CC	Intact		Infectious
102	102-18-TF	Intact		**Not infectious**
186	186-7-TF	Intact		Infectious
198	198-7.2-TF	Intact		Infectious
	198-7.4-TF	Intact		Infectious
	198-7.6-TF	Intact		Infectious
	198-7.8-TF	Intact		Infectious
479	479-11-TF	Defective	***Pol* (**1 bp insertion in RT, a premature stop codon in RNase H)	**Not infectious**
498	498-03-TF	Defective	***Pol (RT)*** (1 pb deletion in reverse transcriptase)	**Not infectious**
499	499-04-TF	Intact		Infectious
527	527-09-TF	Intact		Infectious
	527-20-TF	Intact		Infectious
	527-C12-TF	Intact		Infectious

**Fig. 4:**
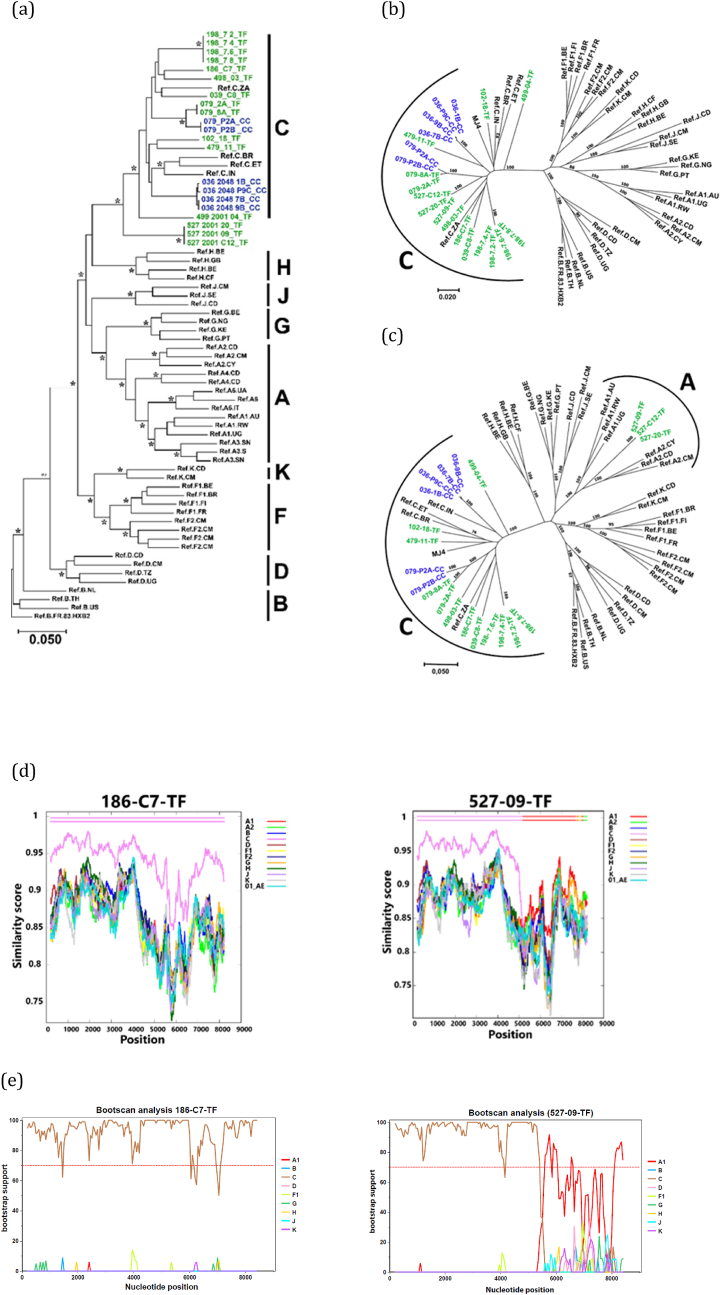
Sequence analysis of the molecular clones. (a) Phylogenetic relationship of the molecular clones. Full-length sequences of generated molecular clones were compared with representative HIV-1 sequences of subtypes A, B, C, D, F, G, H, J and K. A maximum likelihood tree was constructed from nucleotide alignment of full-length HIV-1 genomes. Horizontal branch lengths are drawn to scale with the scale bar representing 0.05 nucleotide substitutions per site. Asterisks along the branches indicate the bootstrap values that support branching, out of a total of 100 resampling. The reference sequences for different HIV-1 subtypes were obtained from the HIV sequence database (database). (b–c) Trees showing the phylogenetic relationship of the molecular clones based on the *gag* (b) and *env* (c) sequences. Maximum likelihood trees were constructed from full-length Gag and Env nucleotide sequences. Major subtypes of HIV-1 group M were used as reference sequences. (d) Plots showing similarity of 186-C7-TF and 527-09-TF to a set of reference subtype genomes. Analysis was performed using recombinant analysis program (RIP). The x-axis indicates the nucleotide positions along the sequence alignment. The y-axis denotes the distance between compared sequences plotted. (e) Subtyping analysis of the genome of IMCs 186-C7-TF and 527-09-TF using Rega subtyping tool (version 3.46). The recombinant pattern was determined by comparing IMCs 186-C7-TF or 527-09-TF sequence with reference sequences of HIV-1 pure subtypes A, B, C, D, F, G, J, K. Bootscan analysis of PID 527 sequence showed that PID 527 is a recombinant of subtypes C and A.

Next, we determined the phylogenetic relationships of the full-length sequences as well as for each individual gene. We assessed full-length genome sequence clustering alongside other HIV-1 group M subtype references. All sequences clustered with the subtype C reference sequences except for the PID 527 clones which formed their own branch away from the subtype C and other HIV-1 group M subtype references. A maximum likelihood tree also showed that clonal sequences from each individual formed a monophyletic cluster (Fig. 4a**)**, indicating that these participants were infected with a single variant**.**

We examined sequence phylogenetic clustering in *gag* and *env*. The *gag* region of generated clones clustered with previous subtype C virus sequences (Fig. 4b**)**, with sequences derived from the same individual forming its own monophyletic branch as observed for full sequences. *Env* sequences revealed a different picture where sequences from PID 527 clustered with subtype A reference sequences (Fig. 4c). We then performed a recombination analysis by submitting the molecular clone sequences to the online recombination identification program (RIP) (http://www.hiv.lanl.gov/content/sequence/RIP/RIP.html) and Rega subtyping tool (https://www.genomedetective.com/app/typingtool/hiv). The recombinant analysis plots revealed a gravitation towards subtype A1 sequences and identified PID 527 as an intersubtype recombinant virus, specifically an AC recombinant (Fig. 4d–e). Individual gene-specific maximum likelihood phylogenetic trees confirmed that PID 527 was infected by an AC recombinant whose genome region from *gag* to *vpr* was subtype C, whereas *vpu*, *nef* and *env* corresponded to subtype A (data not shown).

### 3.4 Replication kinetics of viruses produced by molecular clones in PBMCs

**Fig. 5:**
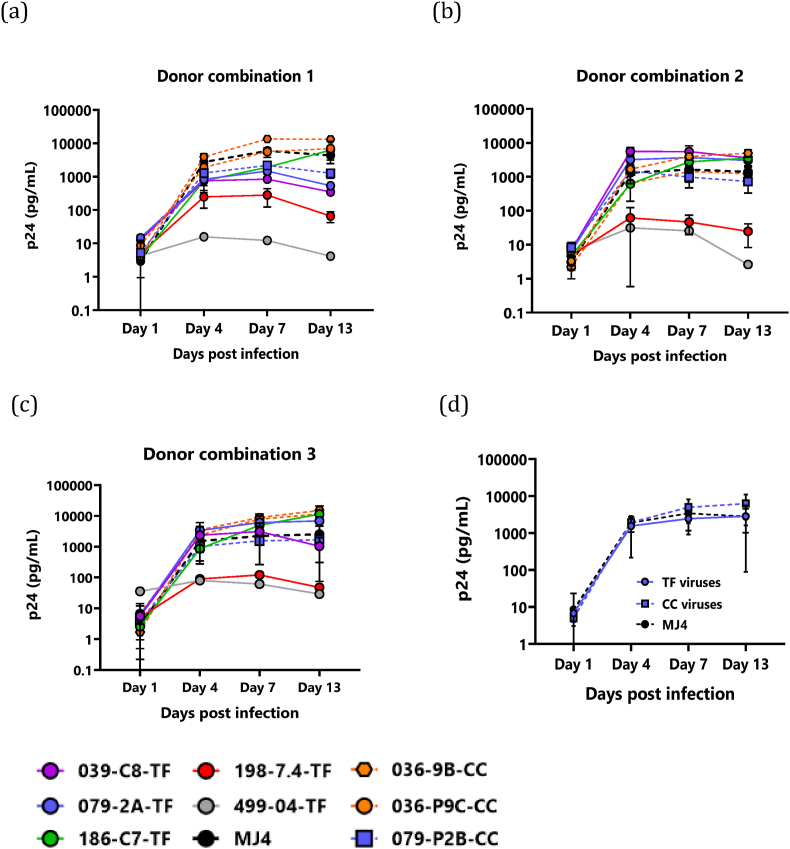
Replication kinetics of viruses in PBMCs from 3 different donor combinations. Activated PBMCs from 3 seronegative donor combinations (a–c) were infected with IMC derived viruses with viral titres equivalent to 20 ng of p24: donor combination 1 - PBMCs from PID 062 and 064 (a); donor combination 2 - PBMCs from PID 044 and 129 (b); and donor combination 3 - PBMCs from PID 151 and 226 (c). The replication kinetics of viruses are shown alongside the prototypic subtype C viral IMC MJ4 (black dashed lines). Values on the y-axis denote pg per ml of p24 and the x-axis days post-infection. T/F viruses are shown in solid lines and chronic-derived viruses are shown in dashed lines. Viral replication was measured using p24 ELISA. (d) Comparison of replication kinetics of 5 T/F and 3 chronic infection viruses is shown. Data points and error bars indicate the means and standard deviations, respectively.

We next examined the growth kinetics of the full-length clones in PBMCs. An amount of virus corresponding to 20 ng of p24 was used to infect PBMCs from 3 different donor combinations, with 2 donors per combination. Replication was assessed by measurement of the p24 antigen concentration in tissue culture supernatant collected on days 1, 4, 7 and 13 after infection. Molecular clones readily proliferated in all three PBMCs donor combinations as indicated by an increase in p24 levels after day 1 (Fig. 5a–c). In PBMC cultures, viral p24 concentrations increased over a 7-day period, with kinetics similar to the MJ4 control virus. Of the 5 T/F-derived viruses tested, 2 viruses (499-04-TF and 198-7.4-TF) were not replication competent in PBMCs, as evidenced by flat curves with p24 concentrations below 200 pg/ml Fig. 5a–c. We observed interpatient heterogeneity with the chronic viruses reaching higher kinetics compared to T/F viruses, however, this was observed only in donor 1. The remaining seven viruses replicated slowly, with kinetics largely comparable to that of the subtype C control virus, MJ4, which generally peaked at p24 antigen concentrations above 1000 pg/ml. Overall, chronic derived viruses peaked at a higher concentration compared to T/F derived viruses: 6263 pg/ml vs 2838 pg/ml Fig. 5d.

### 3.5 V3 loop of both the T/F and chronic derived molecular clones represent a typical R5 consensus amino acid sequence

**Fig. 6:**
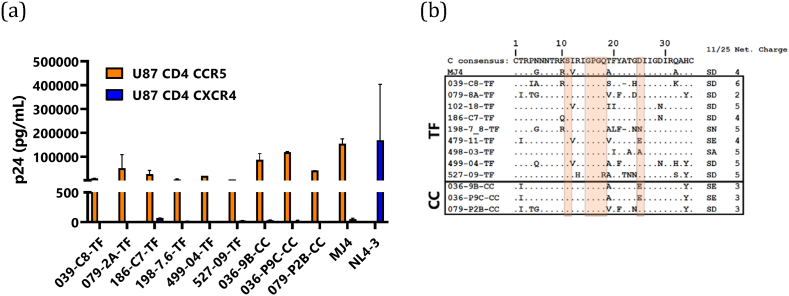
Coreceptor phenotyping of the viral molecular clones (a) Profile of coreceptor use in a U87 CD4 cell line. U87 CD4 cells expressing either coreceptor CCR5 (orange bars) or coreceptor CXCR4 (blue bars) were tested for viral infectivity. The cell lines were infected overnight with viral molecular clones, residual virus was washed off and cells were incubated up to 14 days. Secretion of p24 into the medium was monitored every 3 days using p24 antigen ELISA and data for day 7 are represented here. MJ4 and NL4-3 clones were included as CCR5 and CXCR4 coreceptor using clones, respectively. Infection of each virus variant was measured in duplicates where error bars represent the standard error of the mean (b) Amino acid sequence comparison of the gp120 variable loops. Dots indicate sequence homology and dashes indicate gaps. For the V3 loop, the subtype C consensus sequence is represented at the top and the positions 11 and 25 and the GPGQ motif have been highlighted using open boxes.

Phenotypic characterisation of coreceptor usage was carried out by infecting U87 CD4 cells with expression of either CCR5 or CXCR4. HEK293 T cells derived viral supernatants equivalent to 2000 tissue infectious dose (TCID) was used to infect cells. Infection was assessed by measurement of the p24 antigen concentration in tissue culture supernatant collected on days 1, 7 and 13 after infection. All tested viruses as well as the subtype C reference IMC, MJ4, strictly used chemokine receptor CCR5 as a coreceptor for cell entry. On day 7, generated viruses replicated to high p24 titers in U87 CD4 CCR5, but did not replicate in U87 CD4 CXCR4 cells, strongly indicating preference for CCR5 as a coreceptor for entry. As expected, NL4-3 did not replicate in U87 CD4 CCR5 cells but only in U87 CD4 CXCR4 expressing cells (Fig. 6a).

We then examined the molecular features of the V3 loop amino acid sequences in the Env as the V3 loop significantly influences and determines coreceptor preference of HIV isolates. The V3 loop of the T/F and chronic clones (CC) contained 35 amino acid residues that are representative of CCR5-using subtype C consensus. All the molecular features typical and suggestive of CCR5 use were preserved in the V3 loop of the molecular clones. There was an absence of basic amino acids at position 11 and 25— these positions were occupied by a neutral serine at position 11 and aspartic acid or asparagine with negatively charged side chain at position 25. The crown motif GPGQ which is highly suggestive of CCR5 use was present in all subtype C molecular clones. The V3 loop net charges for the T/F viruses ranged from +2 to +5, indicative of CCR5 use. One V3 loop sequence, 039-C8-TF, had a net charge of +6, which is linked to CXCR4 usage, however, phenotypic analysis showed that this clone used CCR5 and not CXCR4 for virus entry. The V3 loop of 039-C8-TF and 198-7.8-TF contained an amino acid deletion at position 22. Despite amino acid differences from consensus C, molecular features of the V3 loop of the generated molecular clones were suggestive of CCR5 use only, apart from 039-C8-TF with a net charge of +6 (Fig. 6b). Overall, the genotypic analysis of coreceptor usage was largely in agreement with the phenotypic analysis which showed CCR5 use only for all clones. There was a discrepancy between genotypic and phenotypic analysis observed for 1 clone, indicating that genotypic predictions cannot be fully relied on and could benefit greatly from validation with in vitro studies.

### 3.6 Compact envelopes with fewer N-linked glycosylation sites in variable regions are preferentially transmitted

**Fig. 7:**
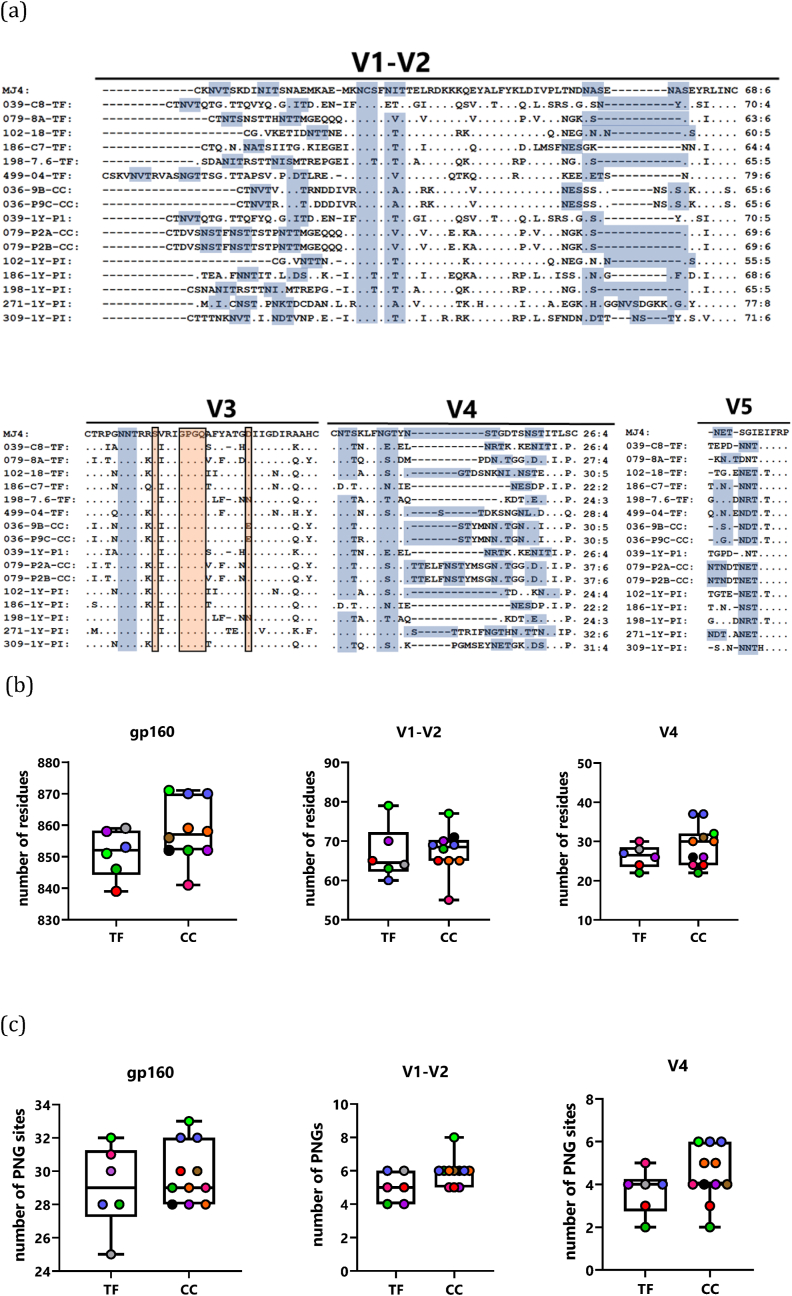
Env amino acid sequence comparison between transmitted/founder and chronic infection clones. (a) The amino acid sequences of transmitted/founder and chronic clone Env variable loops are aligned with the amino acid sequence of the previously described subtype C infectious molecular clone, MJ4. Dots indicate sequence homology and dashes indicate gaps. Putative N-linked glycosylation sites have been highlighted using shaded boxes. Comparisons of the Env amino acid length (b) and potential N-linked glycosylation sites (c) in the env gp160, V1–V2 and V4 regions between transmitted/founder and chronic clones are shown. The medians are indicated by horizontal lines, the boundaries of the boxes indicate the interquartile ranges, and the whiskers display the maximum and minimum values, including the outliers.

As length and glycosylation of Env variable loops have been implicated as selecting features in transmission (Ping et al., 2013; Sagar et al., 2009), we compared these features between the T/F and chronic infection-derived sequences. Firstly, we aligned amino acid sequences of T/F viruses and chronic viruses with the IMC sequence of MJ4 (Fig. 7a). Interestingly, the T/F clone from PID 079 possessed shorter variable regions, V1–V2, V4 and V5, compared to a matched clone derived from 1 year post infection. Inserted amino acid residues in the chronic infection clone created additional putative N-linked glycosylation sites in these loops. This observation is in line with previous reports that the genetic bottleneck at transmission favours variants with fewer potential PNGs and shorter variable loops.

To determine whether transmission selects for viruses with generally shorter envelopes we counted the number of amino acids in the Env gp160 and V1–V2 and V4 regions. Differences in length and glycosylation were observed in the gp160, V1–V2 and V4 domains – the T/F-derived variants tended to have shorter gp160 sequences (median length 852 residues) compared to those from chronic infection (median length 857 residues). The length of the variable domain, V1–V2 and V4 was shorter in T/F-derived variants sequences compared to chronic infection sequences (median residues: 64.50 vs 68.50 and 26.50 vs 30) (Fig. 7b).

Comparison of the PNGs between the T/F and chronic virus sequences revealed that overall, Env gp160 and the V4 domain possessed similar numbers of PNGs, 29 and 4 PNGs in both groups, respectively, while the V1–V2 domain of T/F clones encoded fewer PNGs than the chronic clones (median PNGs: 5 vs 6) (Fig. 7c). Overall, a comparison of T/F and chronic infection Env sequences in our study suggest that reduction in Env length and fewer glycosylation sites in V1–V2 and V4 are associated with transmission.

### 3.7 Differences in type I interferon resistance

Previous studies showed that subtype B T/F viruses were relatively more resistant to IFN-α than chronic viruses or matched 6-month viruses from the same infected individuals, while generally no significant differences were observed for subtype C viruses (Deymier et al., 2015; Fenton-May et al., 2013). We therefore characterized the IMCs generated in this study for their resistance to type I interferons (IFN-α2a and IFN-β).

**Fig. 8:**
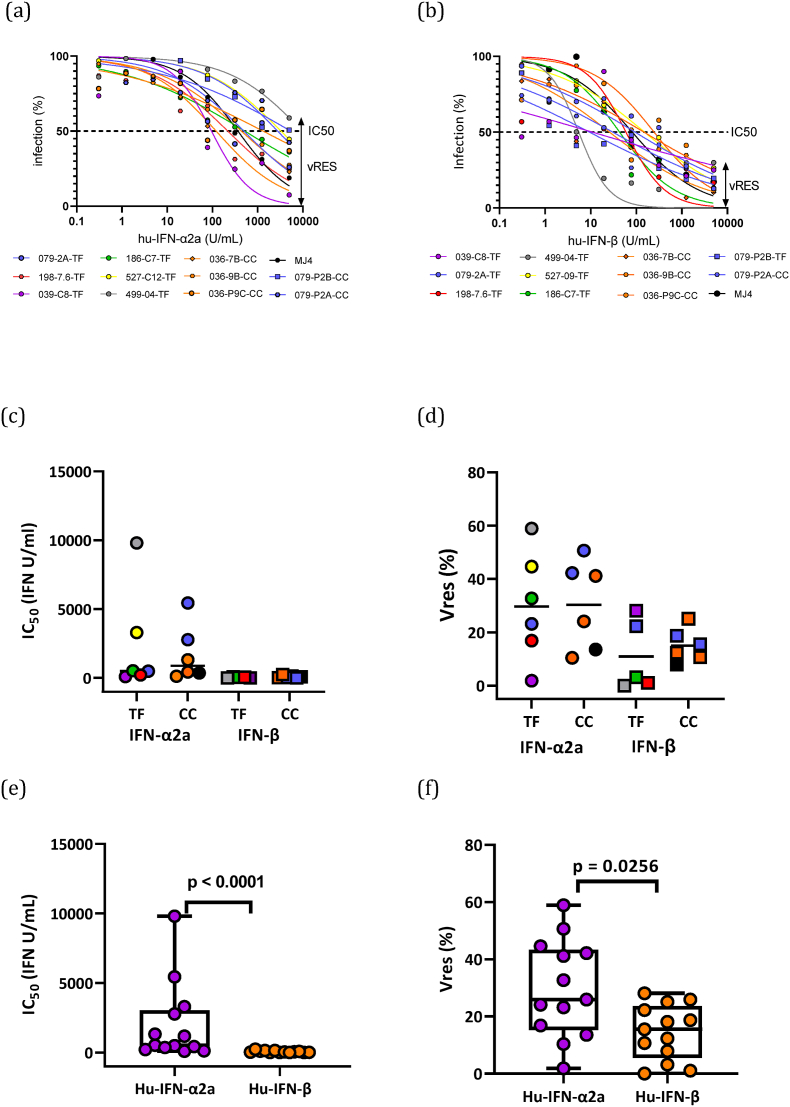
Interferon-α2a and β resistance of transmitted/founder and chronic viruses. (a and b) Dose-response curves for IFN-α2a (a) and IFN-β are shown for transmitted/founder (green) and chronic viruses (blue). The level of infectivity in the presence of IFN (mean luciferase activity, expressed as a percentage of mean luciferase activity in the absence of IFN) is plotted against the IFN concentration and a curve fitted to the data by non-linear regression using a least of squares method. A black broken line indicates the half-maximal inhibitory concentration (IC_50_) and a double arrow, the residual viral infection (Vres) at the highest IFN dose. (c) IFN-α2a (circles) and IFN-β (squares) IC_50_ values of the T/F viruses and chronic viruses. The horizontal lines show the group median IC_50_ values. (d) IFN-α2a (circles) and IFN-β (squares) Vres values of the T/F viruses 039-C8-TF, purple; 079-2A, blue; 186-C7-TF, green; 198 7.6 -TF, red; 499-04-TF, grey; 527-09-TF, yellow) and chronic viruses (036-7B-CC, 036-9B-CC and 036-P9C-CC, orange; 079-P2A-CC and 079-P2B, blue; MJ4, black). The horizontal lines show the group median Vres values. (e) IFN-α2a (purple circles) and IFN-β (orange circles) IC_50_ values of subtype C viruses. (Mann-Whitney's *U* test: p < 0.0001). (f) IFN-α2a (purple circles) and IFN-β (orange circles) Vres values of the subtype C viruses. The horizontal lines show the group median Vres values. (Mann-Whitney's *U* test: p = 0.0256). (039-C8-TF, purple; 079-2A, blue; 186-C7-TF, green; 198 7.6 -TF, red; 499-04-TF, grey; 527-09-TF, yellow) and 6 chronic infection viruses (036-7B-CC, 036-9B-CC and 036-P9C-CC, orange; 079-P2A-CC and 079-P2B; blue).

TZM-bl cells treated with increasing quantities of IFN and infected with equal amounts of virus based on TZM-bl TCID at a MOI of 0.1, were cultured for 48 h. The level of viral infection was measured for each IFN concentration as luciferase activity and expressed as the percentage infection in the absence of IFN, which was set to 100%. This percentage infection was plotted against IFN concentration, and a non-linear regression curve was fitted for each virus. Firstly, we determined the half-maximal inhibitory concentration (IC_50_) of both IFN-α2a and IFN-β for the generated viruses. We measured viral infectivity at the highest IFN dose and expressed this residual infection (Vres) as a percentage of the viral infectivity in the absence of IFN for both IFN-α2a and IFN-β for every virus (Fig. 8a and b).

T/F and chronic viruses exhibited similar sensitivities to both IFN-α2a (median 508.7 vs 881.8 U/ml) and IFN-β (median 43.19 vs 56.27 U/ml) (p (Fig. 8c). We found that T/F-derived viruses displayed different resistance to IFN-α2a and IFN-β when compared to chronic-derived viruses (median: 28 vs 32.6 and 3.3 vs 13.9, respectively)) (Fig. 8d). We did not perform direct statistical comparison between T/F and chronic infection viruses because the chronic infection IMCs were derived from two participants.

Interestingly, the IMCs exhibited a wide range of sensitivities to both IFN-α2a and IFN-β, and viruses were much more sensitive to the IFN-β than IFN-α2a (Fig. 8e), the median IC_50_ was 520.4 U/ml compared to 43.19 U/ml, respectively, p < 0.0001. At the highest dose of IFN-α2a (5000 IU/ml), viruses retained 1.80–58.9% of their respective infectivity (median 25.89%), while at the highest dose of IFN-β (5000 IU/ml), viruses retained 0.01–28.1% of their respective infectivity (median 14.04%) Fig. 7f. Thus, the ability of viruses to infect at the highest IFN dose was 1.1- to 179- fold higher with IFN-α2a compared to IFN-β. These results indicate that IFN-β is more potent at activating viral restriction pathways that act mostly on the early stages of viral replication than IFN-α2a.

## 4 Discussion

HIV-1 subtype C strains continue to dominate the global HIV epidemic suggestive of enhanced transmission potential, although founder effects cannot be fully ruled out. With the limited number of viral variants able to establish infection from a genetically diverse virus population in the transmitting partner (Keele et al., 2008; Tully et al., 2016), selection may play a role in infection establishment. It is evident that HIV-1 encounters a severe genetic bottleneck during transmission, and available data indicate that this is even more stringent during heterosexual FTM (Carlson et al., 2014). The extent to which the transmission process selects for viruses with specific properties is still controversial. Selection during transmission is linked to viral properties including CCR5 tropism (Keele et al., 2008; Roos et al., 1992; Schuitemaker et al., 1992; Wilen et al., 2011), shorter Env variable loops and a loss of PNGs (Chohan et al., 2005; Derdeyn et al., 2004; Liu et al., 2008; Ping et al., 2013; Sagar et al., 2009), increased infectivity, high replication capacity (Parrish et al., 2013) and higher resistance to type I IFN (Fenton-May et al., 2013; Parrish et al., 2013). However, it remains unclear whether these traits are generalizable across the pandemic, as cohorts often differ in the transmission mode and the subtype studied.

To generate useful tools for studies of the transmission bottleneck and investigate selective forces in our setting, we constructed full-length clones of HIV-1 subtype C MTF transmission recipients from nine HIV-1 acutely infected individuals (Fiebig stage I) and two chronically infected individuals (one-year post-infection). We further assessed the genotypic and phenotypic properties between T/F and chronic IMC-derived viruses—viral features including replicative capacity in PBMCs, coreceptor tropism and sensitivity to IFN-α2a and IFN-β.

The molecular clones generated in this study produced infectious viruses following infection of HIV permissive cells. Infection of donor PBMCs with IMC-derived viruses revealed donor variability in replication, with T/F variants replicating relatively poorer in one donor combination compared to 2 other donor combinations. This variability in replication may stem from variations in host factors that enhance or inhibit virus replication. Previous studies have yielded conflicting results on whether T/F variants have higher or lower replicative capacity when compared to chronic infection viruses (Deymier et al., 2015; Oberle et al., 2016; Parrish et al., 2012; Wilen et al., 2011). Moreover, low replication capacity was previously associated with more consensus-like variants— a transmission fitness trait (Deymier et al., 2015; Naidoo et al., 2017; Wright et al., 2010). However, since the IMCs from chronic infection originated from two donors and thus genetically and phenotypically interrelated, our study was unable to explore how T/F variants differed in replication capacity and bias towards consensus sequence compared to chronic infection-derived variants. Overall, the question of phenotypic features that characterize T/F remains unresolved and will require further investigation.

Consistent with previous findings (Chohan et al., 2005; Sagar et al., 2009; Wilen et al., 2011), sequence comparison of the *env* gene revealed the presence of variants with compact envelopes and fewer PNGs in *env* variable regions during transmission. Chronic *env* sequences were bulkier in terms of loop length and PNGs in the V1–V2 and V4 regions. Previously, a study showed that HIV subtype C isolates with compact *env* V1–V2 regions interacted better with target cells, including primary CD4 T cells and monocyte-derived dendritic cells (MDDs), resulting in improved fusion efficiency and envelope incorporation into virions (Cavrois et al., 2014). Therefore, the compact envelope in our study is consistent with the notion of improved viral fusion kinetics and uptake as a potential basis of selection during MTF transmission.

So far, one of the most widely agreed-upon characteristics of T/F viruses is the selection of CCR5 coreceptor tropism during transmission (Keele et al., 2008; Roos et al., 1992; Schuitemaker et al., 1992; Wilen et al., 2011). A recent study, that characterised subtype C Env clones reported a preference of dual tropic viruses that use CCR5 and CXCR6 in 20% of viruses transmitted via MTC (Ashokkumar et al., 2018). However, CXCR6 was not explored in this study. We examined coreceptor usage from a subset of T/F and CC variants. All tested variants, including chronic derived variants, utilised CCR5 as a coreceptor of entry. It has been suggested that the maintenance of CCR5 tropism in subtype C even in advanced stages of infection extends the window of opportunity for subtype C transmission into a new host and facilitates subtype C viruses' relatively high transmissibility, which may underpin its rapid global expansion (Gartner et al., 2020; Tscherning et al., 1998).

Enhanced evasion of the intrinsic immune defence may provide a plausible mechanism for the selective transmission of specific variants. Because innate antiviral cytokines, including IFN-α are induced at the initial sites of HIV-1 replication in the mucosa (Arens and Schoenberger, 2010; Okoye and Picker, 2013), HIV-1 variants that are more resistant to type I IFN may have an advantage during transmission (Iyer et al., 2017; Parrish et al., 2013), however some studies have failed to confirm this finding (Deymier et al., 2015; Oberle et al., 2016). In our study, both T/F and chronic infection viruses displayed variability when tested for sensitivity to IFN-α2a and IFN-β between T/F and chronic viruses, but we eschewed a statistical comparison between these groups due to the inherent bias of chronic infection viruses being generated from only 2 participants. Further studies will be required using genetic tools, such as the ones generated in our study. When comparing IFN-α2a to IFN-β, IFN-β was more potent at blocking viral infectivity in all tested subtype C viruses. Our findings are therefore consistent with a previous study showing that both subtype B and C viruses were more susceptible to IFN-β than IFN-α in CD4 T cells (Parrish et al., 2013).

The generation and initial biological characterization of the subtype C T/F IMCs provide necessary underpinnings for HIV prevention and pathogenesis research to elucidate virus-host interactions during the earliest stages of HIV-1 clinical infection. Therefore, the molecular clones generated here expand the library of T/F virus genomes available for research. Moreover, these reagents can further facilitate a systematic molecular analysis of genome-wide structure and function, and immunological tests of this clinically relevant viral subtype.

We speculate that the viral population bottleneck associated with MTF mucosal transmission is primarily mediated by physical barriers associated with the cervicovaginal and specific virus-host cell interactions necessary for virus infection and replication. Given that the female genital tract undergoes periodic fluctuations in sex hormone concentrations, which affect the architectural and immunological changes in the cervicovaginal tissues and may facilitate HIV acquisition in vaginal epithelium tissue, it is highly likely that less fit viruses are transmitted (Ochiel et al., 2008; Vitali et al., 2017). Defining the viral factors that underpin this fitness may provide additional insight into the biology of mucosal HIV-1 transmission, hence aiding creating of vaccine immunogens.

Our study focused on T/F features that may influence virus entry, overall virus replication characteristics that may impact on subsequent disease progression and the host innate defences that form the first line of defence immediately following infection. The IMCs in our study were derived from a limited number of participants, a study limitation that prevented us from identifying phenotypic features associated with the HIV-1 transmission genetic bottleneck. Moreover, all infection cases in our study were from presumably from male to female transmission, which has previously been associated with a less stringent genetic bottleneck (Carlson et al., 2014). The overall diversity of HIV variants, even during transmission, complicates research on T/F viruses. The T/F and chronic infection viruses generated here, even though derived from a limited number of participants will be a useful resource to enable more detailed comparative and mutagenesis-based studies in future. However, it should be noted that in our study, some of the T/F IMCs were constructed from HIV-1 half genomes amplified by bulk PCR, with the potential that these are recombined genomes that do not exist naturally in vivo. However, these potentially recombined genomes still represent the relatively homogenous variants that establish infection.

## 5 Conclusion

We have generated IMCs of T/F HIV-1 subtype C (Fiebig stage I) from six acutely infected individuals and two chronically infected individuals (one-year post-infection) from KwaZulu-Natal, South Africa, the epicentre of the HIV-1 pandemic. These reagents will be useful in future studies looking for protective factors against HIV-1 clade C viruses. We confirmed that compact Env variable regions and fewer PNGs are a distinguishing characteristic of T/F viruses compared to viruses from chronic infection. CCR5 tropism was a dominant characteristic of the subtype C IMCs. The T/F and chronic infection IMCs were heterogenous in in vitro replicative capacity and resistance to type I interferons, however, the limited number of participants from whom the IMCs were generated, particularly for the chronic infection clones, precluded from firm comparative explorations of the phenotypic characteristics that distinguish T/F from chronic infection viruses. The subtype C IMCs displayed a wide range of sensitivity to both IFN-α2a and IFN-β, and the viruses were much more sensitive to IFN-β than IFN-α2a. The clones generated in this study will be a useful resource for future studies that seek to identify viral factors associated with establishment of infection and subsequent clinical outcomes.

## Funding

This work was supported in part by grants from the 10.13039/100000865Bill and Melinda Gates Foundation (grant numbers OPP1212883 and INV-033558), 10.13039/100005564Gilead Sciences, Inc (Grant ID #00406), the 10.13039/100000866International AIDS Vaccine Initiative (IAVI) (UKZNRSA1001), the 10.13039/100000060NIAID (R37AI067073), and the South African Research Chairs Initiative (grant # 64809). This research was also funded in part by the 10.13039/100010269Wellcome Trust [grant # DEL-15-006] and the UK Foreign, Commonwealth & Development Office, with support from the Developing Excellence in Leadership, Training and Science in Africa (DELTAS Africa) programme, Nairobi, Kenya. For the purpose of open access, the author has applied a CC BY public copyright licence to any Author Accepted Manuscript version arising from this submission.

## CRediT authorship contribution statement

**Bonisile Luthuli:** Investigation, Methodology, Formal analysis, Visualization, Writing – original draft. **Kamini Gounder:** Investigation, Methodology, Writing – review & editing. **Martin J. Deymier:** Investigation, Methodology. **Krista L. Dong:** Writing – review & editing, Cohort development, Resources. **Alejandro B. Balazs:** Methodology, Resources, Writing – review & editing. **Jaclyn K. Mann:** Investigation, Supervision, Writing – review & editing. **Thumbi Ndung'u:** Conceptualization, Funding acquisition, Project administration, Cohort development, Supervision, Validation, Writing – review & editing.

## Declaration of competing interest

The authors declare that none of the authors have any conflicts of interest regarding this study.

## Data Availability

Data will be made available on request.
